# Artificial Intelligence Techniques for Automatic Detection of Peri-implant Marginal Bone Remodeling in Intraoral Radiographs

**DOI:** 10.1007/s10278-023-00880-3

**Published:** 2023-07-19

**Authors:** María Vera, María José Gómez-Silva, Vicente Vera, Clara I. López-González, Ignacio Aliaga, Esther Gascó, Vicente Vera-González, María Pedrera-Canal, Eva Besada-Portas, Gonzalo Pajares

**Affiliations:** 1 Department of Conservative Dentistry and Prostheses, Faculty of Dentistry, Complutense University of Madrid, Madrid, Spain; 2 Department of Computer Architecture and Automation, Faculty of Informatics, Complutense University of Madrid, Madrid, Spain; 3 Department of Software Engineering and Artificial Intelligence, Faculty of Informatics, Complutense University of Madrid, Madrid, Spain; 4 Hospital Clínico San Carlos, Complutense University of Madrid, Madrid, Spain; 5 Instituto de Tecnología del Conocimiento (Institute of Knowledge Technology), Complutense University of Madrid, Madrid, Spain

**Keywords:** Dental X-ray, Image processing, Computer vision, Artificial intelligence, Bone resorption, Peri-implantitis

## Abstract

Peri-implantitis can cause marginal bone remodeling around implants. The aim is to develop an automatic image processing approach based on two artificial intelligence (*AI*) techniques in intraoral (periapical and bitewing) radiographs to assist dentists in determining bone loss. The first is a deep learning (*DL*) object-detector (YOLOv3) to roughly identify (no exact localization is required) two objects: prosthesis (crown) and implant (screw). The second is an image understanding-based (*IU*) process to fine-tune lines on screw edges and to identify significant points (intensity bone changes, intersections between screw and crown). Distances between these points are used to compute bone loss. A total of 2920 radiographs were used for training (50%) and testing (50%) the *DL* process. The *mAP@0.5* metric is used for performance evaluation of *DL* considering periapical/bitewing and screws/crowns in upper and lower jaws, with scores ranging from 0.537 to 0.898 (sufficient because *DL* only needs an approximation). The *IU* performance is assessed with 50% of the testing radiographs through the *t* test statistical method, obtaining *p* values of 0.0106 (line fitting) and 0.0213 (significant point detection). The *IU* performance is satisfactory, as these values are in accordance with the statistical average/standard deviation in pixels for line fitting (2.75/1.01) and for significant point detection (2.63/1.28) according to the expert criteria of dentists, who establish the ground-truth lines and significant points. In conclusion, *AI* methods have good prospects for automatic bone loss detection in intraoral radiographs to assist dental specialists in diagnosing peri-implantitis.

## Introduction

Peri-implantitis is an inflammatory process that causes bone loss [1–3]. According to [4], its prevalence is high among patients with dental implants, 56.6% at the patient level and 27.9% at the implant level, hence the need for early detection. Periapical X-rays can capture individual teeth and the bone around dental implants [5]. The marginal bone loss that these periapical radiographs can show is determinant in evaluating this marginal bone loss and identifying whether it was caused by peri-implantitis. The greater the severity of the disease, the higher the marginal bone loss around the implant. This is a widely reported problem in the scientific and professional dental community that requires the earliest possible detection to undertake the appropriate treatment [6, 7].

Although bone resorption can be easily qualitatively observed from a radiograph with the naked eye by specialists, based on their knowledge, it is not easy to quantitatively determine bone loss and lesion progression over time in terms of severity. This quantification can be computed as the ratio between the bone loss length and the total length of the implant [8]. The manual determination of this ratio and the identification of areas representing bone loss [9] is tedious and very complex. Therefore, the use of an intelligent automatic system for X-ray analysis is essential to assist in assessing accuracy, diagnosis, and treatment to address the effects of peri-implantitis as efficiently and accurately as possible [10].

Advances in the use of artificial intelligence–based systems are becoming more palpable every day in the dentistry field [11], in part due to the efficiency, accuracy, and time savings during diagnostic decision-making tasks, treatment planning, and tracking to determine evolution over time [10, 12, 13].

Thus, an automatic computer-based digital image processing approach is proposed to assist specialists in the diagnosis and quantification of peri-implant marginal bone remodeling. The relevant and critical clinical elements in the intraoral radiographs (periapical and bitewing) are the implants (screws) and prosthesis (crowns) and the surrounding bone, which the specialist identifies intuitively; whereas, for an automatic system, this is a highly complex task (a challenge), requiring extra effort [14], particularly regarding detecting crowns and screws and their relative position, which is the starting point of the imaging process. To achieve a higher comprehension level among different readers, dentists, and AI engineers, hereafter, the pairs of terms implant-screw and crown-prosthesis are used interchangeably, with preference depending on whether one is speaking from a clinical or object detection point of view in computer vision, respectively.

The automatic system is designed so that human expert (dentist) knowledge and reasoning are conveniently mapped and translated in the form of intelligent computer vision–based methods applied to intraoral (periapical and bitewing) radiographs.

The IEEE International Symposium on Biomedical Imaging 2015 [15] identifies dental X-ray image analysis as a grand challenge. From the point of view of digital image processing, the following drawbacks are identified, making automatic procedure development very difficult: (1) existence of natural teeth intermixed with the dental implants, soft tissues, jaw bones, nerves in healthy teeth and three-dimensional (3D) structures mapped into the two-dimensional (2D) image; (2) areas with dark or excessive brightness, due to over- or underexposure and structures with different intensity levels varying from one shot to another, even when using the same capturing device; (3) misarrangement between image receptor, tooth and X-ray beam, producing fake 3D dimensionality; (4) unsharp edges due to out of focus images; and (5) patients with different dental prostheses, missing teeth, or dental irregularities. In addition, more specific drawbacks related to implants and associated prostheses are listed in the Materials section.

Cha et al. [8] proposed a method for peri-implant bone loss measurement in periapical radiographs by using three separate and connected region-based neural networks. The first network identifies the upper or lower jaw, and then, depending on this classification, each of the other two networks is responsible for detecting upper or lower implants and specific landmarks around the screws. The model is a Mask R-CNN [16], where the feature pyramid network is based on ResNet [17]. It uses the feature maps generated in the backbone network of the model to detect landmarks. The box head performs object classification and bounding box regression, and the mask head performs the object segmentation task. The authors add a key point–based detection head with the corresponding training. The aim is to identify six specific and singular key points on the objects (implants) detected by the box head, three on each side of the implant. This is the idea applied for key point human pose estimation, where output heatmaps with significant peaks determine the location of such points during both the training and detection processes in the network [18]. The ground-truth labels for training heatmaps are built by applying 2D Gaussian filtering centered at every key point, i.e., local peaks in such maps. In human pose estimation, key points such as knees, elbows, shoulders, and eyes represent significant points, which differ from the points for detection around the dental implants, i.e., it is not possible to transfer this idea to our problem, which represents a serious handicap. In addition to the previous work, based on the review in [19], we explored the bibliography to verify that no procedures were found combining neural networks and image processing techniques on intraoral radiographs.

With the same purpose as [20], an object detection approach is proposed based on Faster R-CNN [21]. A dataset of images was annotated, considering bounding boxes and remarking key points for determining marginal bone loss assessment. The main drawback to this approach is identifying the key points so that the automatic system based on the object detector can locate them after training. However, it is difficult to locate the beginning and end of areas where marginal bone loss appears.

Sunnetci et al. [22] used two DL neural networks (AlexNet and SqueezeNet) to extract features at the fully connected layer. These features are supplied to different machine learning approaches (support vector machines, K-nearest neighbors or näive Bayes, among others) to determine periodontal bone loss based on the full panoramic radiographs without distinction of dental implants. This approach, based on panoramic radiographs, was also applied in [23]. Panoramic radiographs do not allow bone loss quantification due to lacking detail.

Under such considerations, an automatic process is proposed by applying the following artificial intelligence (*AI*) techniques, making the following contributions: (i) object-based detector YOLOv3 (you only look once) [24] to distinguish between the screw and crown, which form the full implanted structure and to determine whether it is on the upper or lower jaw; (ii) adjustment of a curved line to the edges of the screw; (iii) identification of significant points: marginal bone changes, boundary points between screw and crown; and (iv) relative bone loss measurement, in terms of percentage, with respect to the screw. It should be noted that the division of the upper and lower jaw is of no practical significance in calculating the percentage of bone loss around the implant. This only makes sense to start the process of adjusting the lines and subsequently identify the significant points. These intelligent techniques represent mapping human expertise onto the automated system.

The use of YOLOv3 represents an improvement with respect to the work proposed in [8], based on Mask R-CNN because it does not need the additional key point detection head structure required by the network in [8]. YOLOv3 is also the method applied in [25] only for detecting dental implants, not prosthesis, with success. Therefore, it is a well-proven standard model that can be trained with different types of objects (screws and crowns) without requiring additional image processing and is characterized by its global validity in detecting implants (and prostheses simultaneously as in our approach). Moreover, as reported in [24] on its version three, it is better and stronger than the previous versions of YOLO, with the ability to detect objects at three different scales with nine anchor boxes (three for each scale), i.e., different resolutions of intraoral radiographs can be processed with a sufficient guarantee. This represents a second improvement with respect to Mask R-CNN applied in [8]. In addition, YOLOv3 requires only one network instead of the three used by [8].

**Fig. 1:**
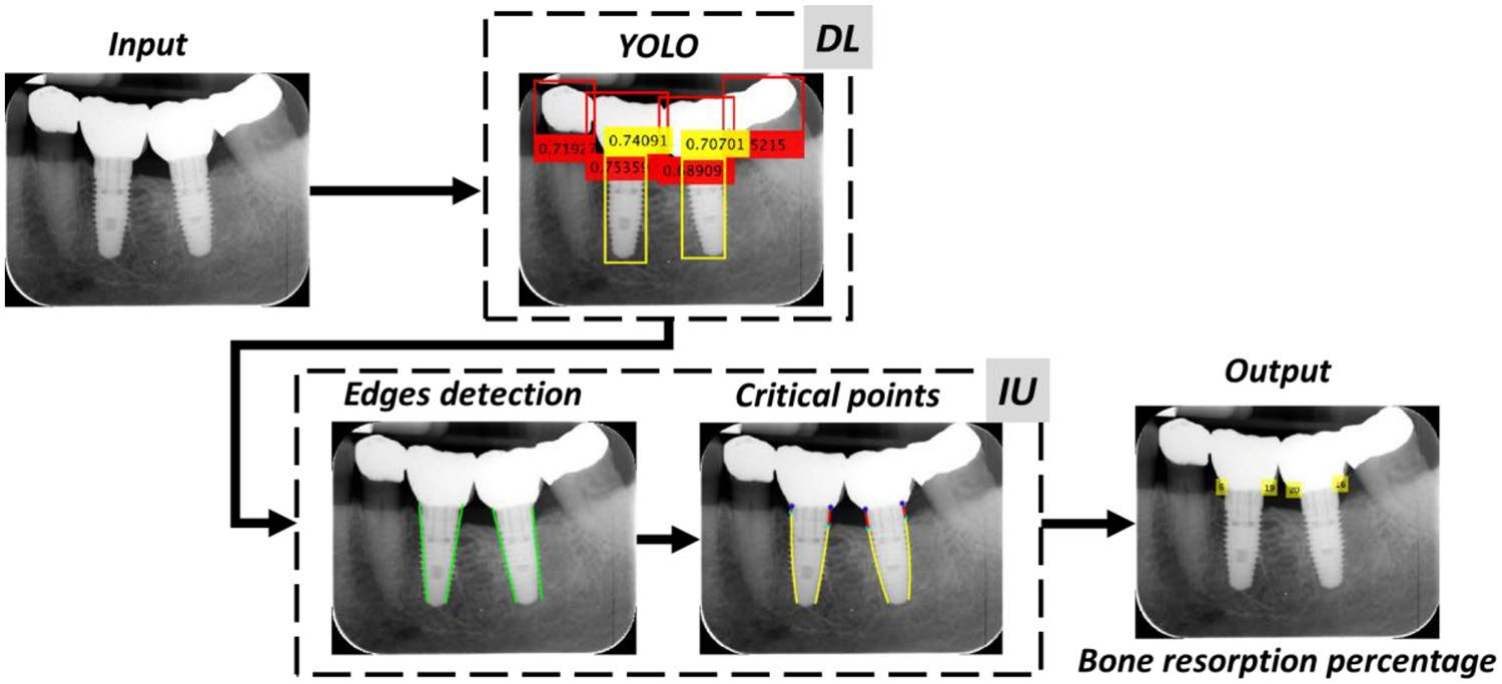
Outline of the full procedure

Two main levels of intelligent processes are considered and implemented in this work. Figure 1 shows the outline of the complete procedure:

- *Deep Learning* (*DL*): DL is the first step of the intelligent system, where both screws and crowns are the objects detected by YOLOv3 in the deep learning context, locating them in the intraoral radiographs.
- *Deep image understanding* (*IU*): IU is the sequence of advanced image processing and computer vision methods and techniques to extract the underlying embedded knowledge in intraoral raw radiographs, sometimes hidden for the human analyst, to measure marginal bone loss or bone remodeling.

In summary, *DL* with a unique network detects objects (crowns and screws) and their relative positions without the need to obtain high precision, thus avoiding the detection of significant points at this stage. Then, computer vision–based processes (*IU*) perform a refinement to identify the significant points, which allows us to quantify the marginal bone loss. Considering this information, Table 1 shows the advantages and shortcomings of these techniques compared to our approach.

**Table 1:** Summary of comparisons of previous and proposed methods

References	Description	Shortcomings	Remodelling bone loss quantification
[11]	Use a Mask R-CNN object detector with three networks to extract six landmarks (significant points) from feature maps	The feature maps must be obtained with levels of activation, just on the six landmarks to be detected	Yes
[16]	Use a Faster R-CNN object detector to identify specific key points	Critical specific key points must be located just at the beginning and end of the marginal bone loss areas	Yes
[22, 23]	Use different machine learning strategies to classify bone loss or not. The inputs are the features extracted from the fully connected layer in Convolutional Neural Networks	The classification is based on panoramic radiographs. It is not possible to quantify the marginal bone loss due to the lack of detail in this type of images	No
Ours	DL: use YOLOv3 object detector (unique network) to detect implant structures. IU: then apply robust techniques computer vision-based techniques to identify significant points without the need to use YOLOv3 activation maps	When the implant and surrounding tissues display low intensity contrast on the radiographs, or the marginal bone remodelling is non-existent, the significant point detection fails	Yes

## Materials and Methods

### Materials

This study was carried out on a dataset of 2336 dental intraoral digital radiographs acquired with a KaVo™ FOCUS. FOCUS™ intraoral X-ray IEC 60601–1-2e machine operating at 70 kV, 7 mAs and 0,100 s of exposure with a short cone, round SSD 229 mm/9″, Ø60 mm under adult mode. Ten percent of the total radiographs (234) come from 62 patients, so for each patient, there are between 4 and 6 captures with different degrees of bone resorption (due to disease progression over time), taken on different days spaced at least 6 months apart. Those belonging to the same patient have different intensity features depending on the operating conditions of the device on the day of capture. There is not necessarily a correlation between those of the same patient. Structural similarity only exists in the case that each patient keeps the same teeth. Therefore, this does not affect the training and testing processes. A total of 350 radiographs (approx. 15%) belonged to the bitewing type, and the rest belonged to the periapical type (approx. 85%). Within these proportions, approximately 73% are implants in the lower jaw. In all cases, we used the Rinn XCP (“extension cone paralleling technique”). This film holder is used in intraoral dental X-rays and is specifically recommended in both types, periapical and bitewing, to standardize the technique. The election of the type of Rinn XCP and the X-ray was performed according to the implant location, and adjacent teeth and by considering the presence or absence of surrounding and antagonist teeth.

The images were stored in JPEG format with an image resolution of 410(*H*) × 340(*V*) pixels as intensity images with sufficient quality. The quality is determined by the intensity contrast between the implants and the surrounding tissues. Its quantification is established by considering the bounding boxes obtained in the *DL* process as a function of the standard deviation relative to the intensity, as explained later in the Evaluation section. Regarding the calibration process, we considered implant sizes with lengths of 10, 13 and 15 mm (40 lengths of each), and it was determined that, on average, the equivalence between one pixel in the vertical (*V*) direction is approximately 0.063 mm. The measures in pixels on the images were determined by all clinical authors, agreed upon, and pooled with the interactive distance measurement function of MATLAB [26].

Additionally, we randomly augmented the initial dataset by 25% for each type (bitewing and periapical) with the identical resolution, resulting in a total of 2920 images. Image enhancement and rotations with angles varying ± 10° were applied [27]. The purpose of this augmentation process was to better verify the robustness of the proposed approach by introducing radiometric (intensity changes) and geometric (by rotation) modifications to add more contrast and different positions on the dental pieces. This modification improved the training process involved in the *DL* level since it receives more diverse images. It was also possible to verify that the performance at the *IU* level was preserved despite the augmentation process.

Of these 2920 images, 50% were randomly selected for training the YOLOv3 model at the *DL* level, resulting in a total of 1460 images, keeping the proportion of 15/85% between bitewing and periapical. The other remaining 50% of images (1460) were used for testing at both levels, i.e., *DL* and *IU*. The *IU* level did not involve any training, although those images used for training YOLOv3 did not participate in the *IU* testing process.

An important aspect to consider here, despite the relatively small number of images used for training at the DL level and considering that deep learning models require a large number of images, is the high performance of the YOLOv3 method for detecting crowns and screws as we analyze later in the Evaluation section.

The images were processed by MATLAB R2023 [26] using an Intel(R) Core (TM) i7 2.0 GHz processor, 16 GB RAM and Windows 10 Pro operating system (64-bits).

These images contained different structures and morphologies that can be summarized as follows:

- Different sizes and inclinations of dental implants in the intraoral radiographs. Thus, no fixed sizes and orientations must be expected.
- Different intensity levels in different parts of the images, including dental implants that surround them, even in the same image.
- Curved edges of implants with the corresponding threads.
- Irregular texture patterns in marginal bone loss.
- The implant and crown limits display higher intensity levels than the rest of the structures.
- The marginal bone limits around implants could present irregular shapes with low or very low gray intensity levels, making its processing difficult.

### Methods: Intelligent Image Processing

The previous considerations demonstrate the complexity of the images, although some of them, such as (e) and (f), favor some parts of the automatic detection process and all together allow the design of the proposed intelligent and automatic strategy.

#### Detecting Implants as Objects

As mentioned before, YOLOv3 is proposed to detect and locate the screwed part and the crown part, which are the two types of objects to be detected. It also allows us to determine the relative position of both parts with the aim of identifying whether the implant belongs to the upper or lower jaw. Some approaches based on convolutional neural networks (CNNs) can be found in [28]. Lee and Jeong [29] applied a pretrained deep CNN architecture (GoogLeNet Inception-v3) to classify different models of the implant body in one of the three classes studied, including periapical radiographs. This is also the line proposed in [30], where VGG-16 and VGG-19 models were compared against the model of the authors themselves. In this regard, the main drawback of these approaches is to determine the location of the screw for its automatic classification. Once the region of interest is defined, their bounding boxes can be supplied to the network.

Thus, YOLOv3 is trained with these two kinds of objects so that during the detection process, both parts can be differentiated and isolated. This means that two classes are to be considered, i.e., they guide the definition of the three loss functions involved (box, object, and class), particularly the one belonging to the classification loss.

**Fig. 2:**
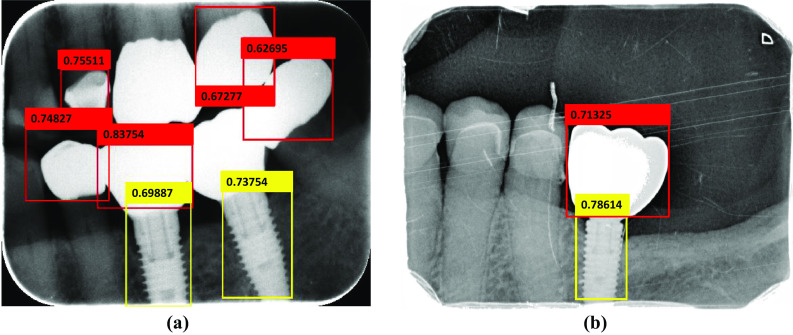
Examples of intraoral radiographs with implants in the lower jaw

The procedure defines the *DL* part introduced in Fig. 1, and it is established as follows, where Fig. 2 serves as a guide for its description:

- Let *I* be the input image to be processed. By applying YOLOv3, two kinds of bounding boxes are obtained, those defining the crowns (red boxes in Fig. 2) and the screws (yellow boxes in Fig. 2). It is well known that each bounding box is defined with its upper left corner position (*x*,*y*), width (*w*) and height (*h*). Moreover, each box contains the confidence score, ranging in [0,1], indicating the objectness, i.e., it says how likely the box is to contain the specified object. We established that this value must be greater than 0.5, which is the intermediate value between null (0) and full (1) detection. As mentioned before, the images used in our experiments are of size 410 × 340 pixels, labeled with their corresponding bounding boxes. To verify the robustness of the *DL* process, we used a subset of 115, again with 15% bitewing and 85% periapical radiographs with sizes of 1164 × 876 and 1602 × 1230 pixels (H × V), respectively, and different from those selected for testing with dimensions of 410 × 340. These images are resized to 410 × 340 pixels by applying bicubic interpolation. Figure 2 displays two illustrative examples that represent these images with their detected bounding boxes and scores. In the periapical radiography in (*a*), five crowns were detected, and two others were not; in the bitewing radiography in (*b*), the unique existing crown was identified. All screws were detected in both images. In this subset, the success rate in detecting crowns and screws is 82% and 93%, respectively, and in 98% of these images, at least one crown and one screw belonging to the same implant are always detected, which is an acceptable result.
- Considering all bounding box centers, we determine if they are implants located in the lower or upper jaw. Considering that the upper left corner of each image represents the origin of the coordinates and that the *y*-coordinate grows downward, we compute the mean value of the *y*-coordinates of each category, *y*_*c*_ (crown) and *y*_*s*_ (screw), and if *y*_*c*_ < *y*_*s*_*,* the implants are in the lower jaw and vice versa. Implants located in both jaws were not considered in the images analyzed. Figures 2a, b and 9b–d display implants in the lower jaw, and Fig. 9a, e, f displays three implants in the upper jaw.

Regarding both images in Fig. 2, extending it to other images in the set, we can see that some parts of the screws are not visible in the two types of intraoral radiographs. Although this fact does not prevent further processing, it is not a problem when the percentage of bone resorption is to be calculated with respect to the total length of the screw. The length in millimeters of the implant is always known. Once the X-ray acquisition device is calibrated by establishing the correspondence between millimeters and pixels (Materials section), the relative and absolute degree of deterioration is immediate. Obviously, the best situation to determine the percentage is to visualize the full length of the screw without occlusions, although the proposed approach was designed to detect critical points where significantly deteriorated areas are identified without such requirements and, therefore, even with missing parts.

#### Detecting Implant Boundaries

**Fig. 3:**
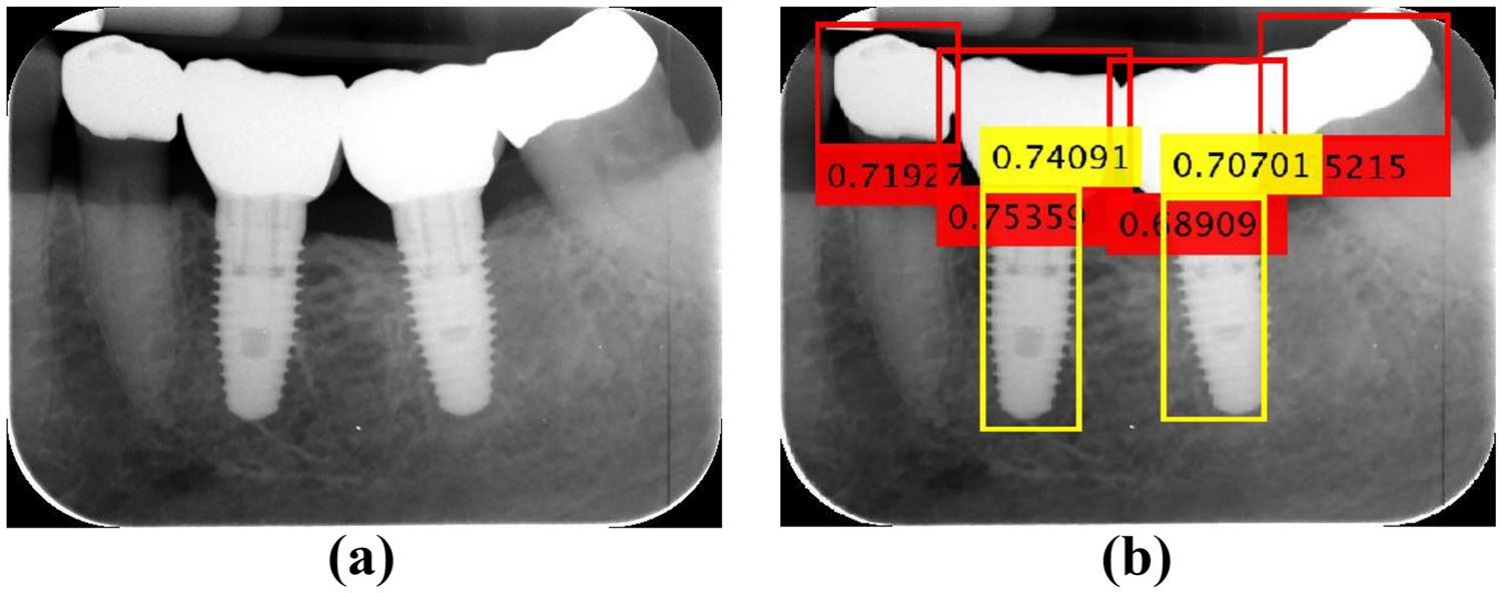
Periapical radiography: **a** original; **b** detected objects (crowns and screws)

Once objects have been detected, the next step is focused on the screws to identify their boundaries and to fit the best line (curve), to end with the computation of the percentage relating to bone remodeling. This is the *IU* process in Fig. 1, which is illustrated based on the periapical image displayed in Fig. 3a. Figure 3b displays the detected objects by YOLOv3. Here, all crowns and screws have been detected with their confidence scores, i.e., all above 0.5. In this case, implants belonged to the lower jaw, according to the process described above.

To validate a crown bounding box, it must be spatially located above or below a bounding box associated with a screw, depending on whether it is a lower or upper jaw implant. This is verified by extending the bounding box of the screw toward the bounding box of the crown in such a way that an overlap, with respect to the crown bounding box, of more than 30% is achieved.

##### Step 1 (Edge Extraction)

**Fig. 4:**
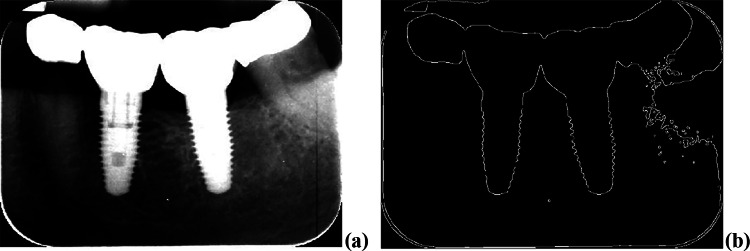
**a** Enhanced image; **b** edges detected by Canny

From the original image, Fig. 3a, image enhancement is applied to increase the differences in gray levels between the artificial parts, which contain the objects to be detected, and the rest of the image. For this purpose, a gamma-based (with γ = 1.5) adjustment is applied [31]. This achieves that the input image levels are weighted toward darker output values, while the artificial parts, with high incoming intensity gray levels, are shifted toward brighter values. The Canny [32] edge detector is applied to the resulting image, Fig. 4a, providing the edges displayed in Fig. 4b.

##### Step 2 (Edge Description)

**Fig. 5:**
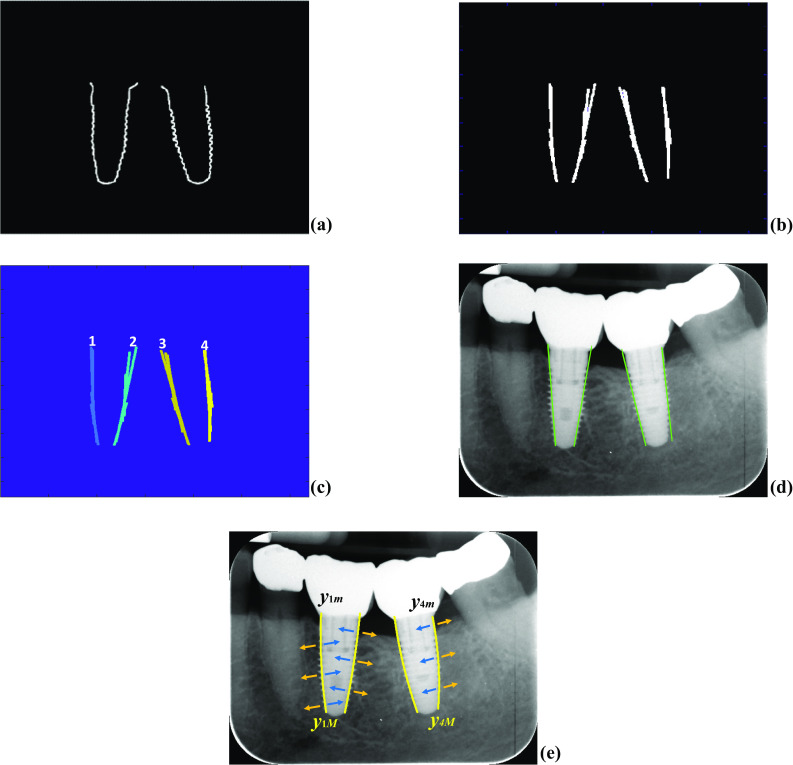
**a** Selecting edges inside the bounding boxes; **b** detecting edge segments; **c** grouping and labeling edge segments; **d** adjusted straight lines; **e** adjusted curved lines

The next step is to select the binarized edges detected by Canny within the bounding boxes corresponding to the objects classified by YOLOv3 as screws. Considering the sizes of the bounding box, it is expanded by 10% in all dimensions to ensure that all edges are inside. On the edge points thus selected, a mathematical operation of dilation is applied [31], with a structured element of 3 × 3, with the aim of expanding the lines that form the edges. In this way, the existence of these edges is emphasized while filling small gaps between them, facilitating the next process in the sense that it is intended to identify continuous and joined edges. The result of this process is displayed in Fig. 5a. This is followed by the process of describing the edges associated with the implanted screws, which is the input for the Hough transform [33–35]. This transform is designed with resolutions of 1 pixel and 1° in the accumulator space in polar coordinates,

1 $$\rho = x\cos\,\theta + y\sin\,\theta$$

where *ρ* is the distance of the straight line at the origin and *θ* is the angle that forms the normal with the *x*-axis. To convert the Hough parameters (*ρ,θ*) to the parameter space of the image (slope, intercept), the following relations were used:

2 $$m = - \frac{\cos\,\theta }{{\sin\,\theta }}, \; d = \frac{\rho }{\sin\,\theta }$$

where *m* is the slope of the straight line and *d* is the intercept.

Sixteen peaks are identified in the Hough polar space, which determine the corresponding *m* and *d* parameters associated with 16 straight lines adjusted to the edges. The goal is to fit several piecewise line segments for the best description of the curved edges. This minimizes the effect of some adverse structures, such as edge protrusions seen at the top of the edges, which is detrimental when trying to fit a unique line. Segments with slopes tending toward the horizontal are excluded since the target edges to be described are oriented toward the vertical. For this purpose, segments with slopes less than 30° are discarded. Figure 5b displays the segments extracted and associated with the four edges. Then, we proceed to isolate groups of segments to fit a unique line on the cloud of points that define the groupings of the segments. Figure 5c displays the grouping of such lines; each group is identified by applying region labeling [35]. Each labeled region is expanded horizontally by a morphological dilation operation with a structural element of 1 × 5. Each labeled and expanded region is intersected with the edges belonging to the bounding boxes, in this example, the ones displayed in Fig. 5a. This allows the isolation of labeled pixel alignments, as displayed in Fig. 5c, on which groups of labels are identified; in this example, four groups (1, 2, 3 and 4) are marked. For each group of labels, two least squared regression-based polynomial settings are applied. The first adjusts a straight line, as displayed in Fig. 5d, and the second is to adjust a curve, i.e., a polynomial of degree equal to or greater than two [36]. In all our experiments, we verified that a degree of two suffices (this is justified according to the experimental results explained in the Results and Discussion section). This means that the polynomial is defined as follows: *x* = *a* + *by* + *cy*^2^, where the independent variable is expressed as *y* and *a*, *b* and *c* are the coefficients estimated by regression. For each grouping of pixels, the points with the maximum and minimum values of component *y* are identified, *y*_*iM*_ and *y*_*im*_, where *i* represents the number of the group. Figure 5e displays the four curves adjusted for the four edges available together with *y*_1*M*_, *y*_4*M*_, *y*_1*m*_ and *y*_4*m*_ for illustrative purposes.

##### Step 3 (Computing Critical Points)

Once straight and curved lines have been adjusted, we are now able to search for critical/significant points along the edges defined by the screws. These points allow us to determine the bone resorption measurements of the structures associated with the soft and hard tissues (mandibular bone). For implants in lower jaws, the starting points are *y*_*iM*_ and the end points *y*_*im*_ and vice versa in the upper jaws. For illustrative purposes, we use the group of pixels labeled with 4, Fig. 5c, e. Therefore, starting at *y*_4*M*_ and progressing to *y*_4*m*_ until reaching it, in steps of one unit, we compute the corresponding values of the *x*-coordinate according to the estimated polynomial expression defined above. Therefore, for each *x* value, we trace a perpendicular line to the corresponding straight line associated with this edge, the green line in Fig. 5d, and following this perpendicular line, we read intensity gray values on both sides of the straight line in the perpendicular direction (blue and orange arrows, Fig. 5e). Ten is the number of pixels set for the expansion along this perpendicular direction. This value was established by trial and error, representing approximately 2.5% of the horizontal dimension (*H* = 410 pixels) of the images. Therefore, this percentage value can be used as appropriate for datasets with images of different dimensions. The reading of these intensity values begins several pixels away from the original straight line to ensure that the implant structure is being scanned against the surrounding tissues. The idea of this process is to determine the intensity contrast on both sides of the straight line so that when there is a significant change in the contrast difference, it is a clue that there is a transition between tissues, expressed as changes in intensity values, and therefore, the transition from a healthy area to a damaged area of the tissue. The contrast sign allows us to determine on which side the implanted part is. If we compute the difference (*D*) between the average intensity levels of the left part (*L*) and the right part (*R*), this difference will be negative for the two left curved lines fitted with each implant and positive for the other two right curved lines, Fig. 5e. Traversing each curved line from the starting point to the end point and for each position (*y*), we apply the following process to obtain an accumulated (*Cum*) vector across all positions:

**Table:** 

*from starting* point to *end* point, each point indexed by *y*, do:
* D* = *L* − *R*
* if D* < 0; *Cum*(*y*) = *L*
* else Cum*(*y*) = *R;*
* end*
*end*

**Fig. 6:**
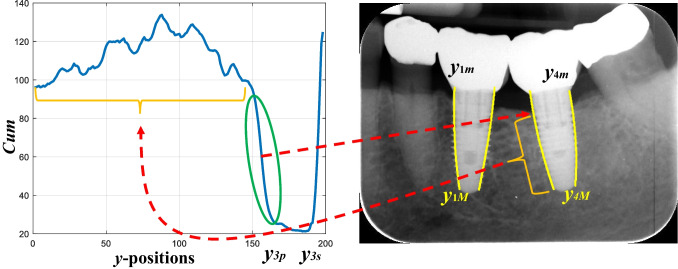
*Cum* profile and position of two critical points

For each *Cum* vector, we compute three points at three samples at which the profile changes abruptly according to the statistical mean value of *Cum*. For illustrative purposes, Fig. 6 displays the profile (blue line) corresponding to *Cum* computed in the curved line of Group 3 in Fig. 5. From the starting point, identified as zero* y*-position at the profile, *Cum* values are positioned at levels above the value 100 and higher until reaching the abrupt change that occurs at the *y*-position of 155 (*y*_3*p*_), just where a significant decrease in intensity levels appears, which corresponds to the starting point of tissue degradation. This is the first critical point for this curved line, located at *y*_3*f*_ = *y*_3*M*_ – *y*_3*p*_ with *x*_3*f*_ = *a* + *by*_3*f*_ + *c*(*y*_3*f*_)^2^. At this point, we compute five *Cum R* values toward the direction of the end point, and these values are averaged to obtain *R*_*av*_, which is a reference intensity level.

**Fig. 7:**
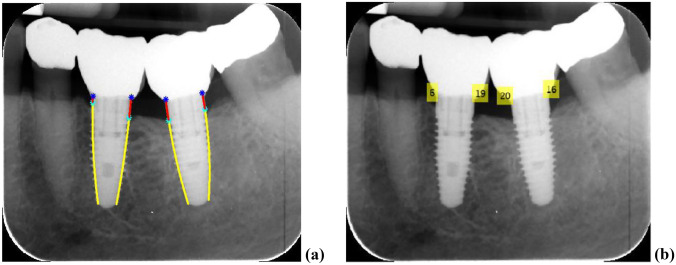
**a** Critical points; **b** percentage of bone resorption

We continue exploring the curved line only until entry into the crown is detected. Continuing with the example of the same curved line, such input is detected when the average intensity profile provided by the new positions of *Cum* from the critical point reaches high intensity values corresponding to the radiographic image of the crown. During the previous process, *Cum* values were computed with the average intensity levels of the left part (*L*), but now, the computation is based on the right part (*R*), guaranteeing full entry into the crown. Therefore, after five explorations with *R* values, all greater than *R*_*av*_*,* we determine that the crown is entered, and therefore, the identification and position of the second critical point, whose *y*-coordinate is taken as that corresponding to the first intensity change in the five explorations considered. In the example, it is *y*_3*s*_ with *x*_3*s*_ = *a* + *by*_3*s*_ + *c*(*y*_3*s*_)^2^. Figure 7a displays the eight critical points detected on this periapical radiograph. The first critical points are marked with cyan asterisks and the second ones with blue asterisks. The piece of each curved line between both critical points determines the degree of bone resorption, and its length with respect to the length of the corresponding curved line determines the percentage of bone resorption. The position of the second critical point can be confirmed by verifying that its position is inside a bounding box defining crowns. Although this verification has not been necessary in our experiments, it is possible that it should be considered in the future. Figure 7b displays the six values, in pixels, obtained for this image, where we can see that the maximum and minimum levels achieve 20% and 6% bone resorption, matching qualitatively with what is visually observed.

## Results and Discussion

### Dataset: Image Description

**Fig. 8:**
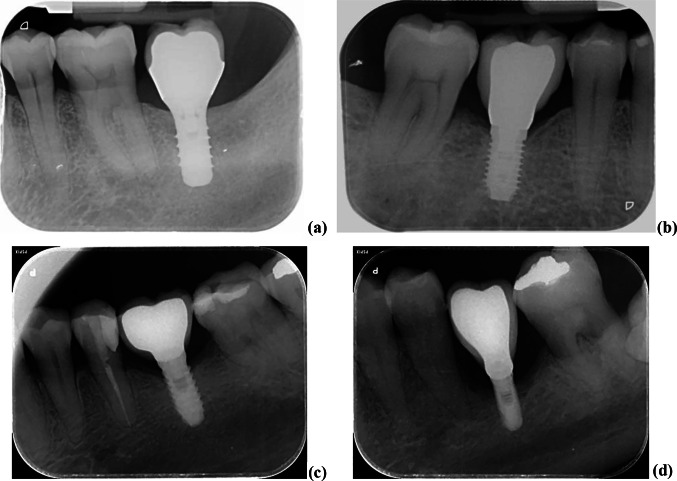
Levels of bone resorption: **a** normal; **b** early; **c** moderate; **d** severe

A total of 2920 (original and augmented) images were analyzed, all captured as described in the Materials section and anonymized under the ethical procedures established by our institution. Each image contains a particular and peculiar morphology with different singularities and features, summarized as follows (see Figs. 2 and 3 for reference): (*a*) a different number of implants; (*b*) each implant has an associated prosthesis, although conversely, there may be artificial structures that resemble a prosthesis without these being associated with the implants; (*c*) upper or lower implants, each one under different inclinations with respect to an imaginary vertical orientation. According to [12], the images contain different levels of bone resorption, depending on the ratio between the bone loss and the total implant length: normal (≤ 10%), including null impact, early (10–25%), moderate (25–50%), and severe (≥ 50%). The measures were carried out by all clinical authors, agreed upon, and pooled with the interactive distance measurement function of MATLAB [26] on each image. Figure 8 contains illustrative periapical examples of each of them, i.e., (*a*) normal, (*b*) early, (*c*) moderate and (*d*) severe.

### Evaluation

To assess the proposed approach, we designed the following tests for performance:

#### YOLOv3: Detection of Screws and Crowns

As mentioned in the Materials section, from the 2920 images, we use 1460 images during the training phase of the YOLOv3 model (1314 for training and 146 for validation, i.e., 90% and 10%, respectively), and the remaining 1460 images are used for testing. All of them are conveniently labeled with the Image Labeler Application of MATLAB [26] to obtain the network model parameters derived from the training and the metrics described below during classification.

Intersection over union (*IoU*), i.e., the Jaccard index [37], is the basic metric used for determining the degree of matching between the predicted and ground-truth bounding boxes associated with each object (screw, crown). From the *IoU* and considering an objectness score greater than 0.5, the well-known *mAP@0.5* metric is used for assessing the YOLOv3 performance. Table 2 displays the *mAP@0.5* values obtained in our experiments with the testing data separated by screws and crowns in periapical and bitewing radiographs, distinguishing between upper and lower implants.

**Table 2:** *mAP@0.5* values for objects screws and crowns as lower and upper implants in periapical and bitewing radiographs

*Jaw*	*Periapical*		*Bitewing*	
	*Screws*	*Crowns*	*Screws*	*Crowns*
*Upper*	0.790	0.685	0.743	0.537
*Lower*	0.898	0.754	0.744	0.630

This validation is only related to the process of detecting clinical structures of interest (crowns and screws) within the *DL* process (Fig. 1); therefore, its relevance is restricted to such a process but not from a strictly clinical point of view. However, it is worth noting that any failure at this stage will have a negative impact on the clinical bone resorption computation.

Higher *mAP@0.5* values indicate better performance. Therefore, from the results in Table 2, we can see that the best performance is achieved in screws as lower jaw implants, particularly in periapical and not so much in bitewing. Regarding crowns, it is also in the lower jaw where the best results are obtained. This situation is determined exclusively by the type of images, which contain a greater distribution and distinction of the lower jaw parts versus the upper jaw parts. Regarding the best performance of screws against crowns, we verified that this is because they appear more separated in the images than crowns that are much more grouped in both lower and upper jaws, being more difficult to distinguish them at the individual level. These results correspond with those obtained in [8] and, therefore, in the same range of values. The aim is to identify the clinically relevant parts (crowns and screws) to guide subsequent *IU* processing without the need to achieve maximum precision at the *DL* stage, as this responsibility is entrusted to *IU* processing. Therefore, achieving values such as those obtained in [7] is considered acceptable.

At this point, it is necessary to discuss the number of images used for training the YOLOv3 model at the *DL* level. This number was 1460, generating a total of 6124 objects labeled crowns and 2988 labeled implants, with their corresponding anchor boxes, making a total of 9112. The network was trained from scratch instead of applying the concept of transfer learning where the models were pretrained, usually with color images, which differ from the gray images used in this work.

The number of labeled objects apparently represents a relatively low number in the context of DL. However, two things concerning the proposed approach should be considered in this regard:

- There are YOLOv3-based models in the field of medical imaging [38], specifically of the CT type and, therefore, gray images, such as those used in this work, with a number of objects per class equivalent to that used in this work, achieving high performance. In [18], a similar number of samples with respect to the order of magnitude is used for testing the object detector based on the Faster R-CNN model.
- The main goal is to describe the screw edges guided by their bounding boxes but not the exact detection of screws. Consequently, high accuracy of such a detection is not mandatory, and therefore, the use of a high number of images and an equivalently high number of objects is not decisive.

**Fig. 9:**
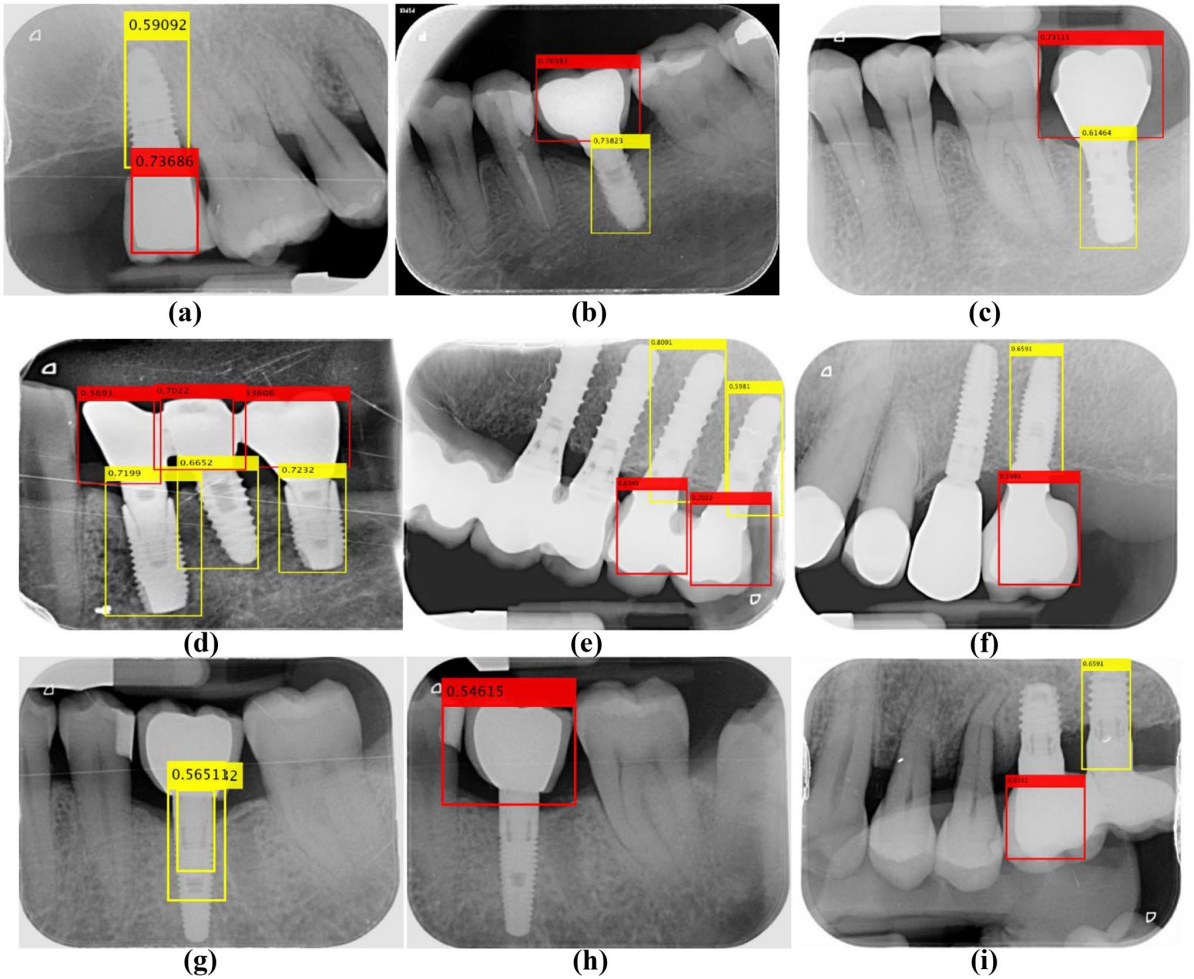
Bounding box detection: **a**–**f** valid; **g**–**i** not valid

During the testing process of the *DL* approach (classification phase with YOLOv3), the following considerations need to be made based on the illustrative examples displayed in Fig. 9:

- Regarding bounding box detection, images (*a*) to (*f*) display valid detections. At least one correct screw bounding box is detected since there exists a crown bounding box that receives the screw bounding box with at least 30% overlap, as described in the detecting implant boundary section. In contrast, from (*g*) to (*i*), there are no valid bounding box associations since either there is only a single bounding box or there is no such overlapping. In all cases, valid and not valid, all bounding boxes for both classes achieve scores above 0.5, as needed. Only valid detections are transferred to the next process (*IU*) to extract the boundaries. In our experiments, considering the 1460 images used for testing, 66 of them were excluded, making a total of 1394 images used for the *IU* testing process.
- Regarding the intensity levels, which represent the implants and crowns in relation to the rest of the bone structures or tissues, it can be inferred that correct identifications are obtained regardless of whether there are differentiated contrasts. The contrast is determined as a function of the standard deviation (*σ*) of the intensity levels within the bounding boxes. In the set of images analyzed, it is determined that high contrast values are obtained with *σ*_*max*_ ≈ 98, while the minimum is *σ*_*min*_ = 0. Normalizing the *σ* values to the range [0,1], we can determine that 0.4 is an appropriate threshold. The *σ* values for (*a*), (*c*) and (*f*) are 0.19, 0.23 and 0.38, respectively (lower than the threshold), i.e., low contrast. In (*b*), (*d*) and (*e*), the *σ* values are 0.68, 0.79 and 0.63, i.e., high contrast.

#### Fitting Lines to Screw Edges

We designed a specific testing strategy to assess the performance in adjusting lines along the screws. To analyze this fit, it is unnecessary to distinguish between the upper and lower jaw or between the anterior and posterior teeth, as only the fit along the edges of each screw is of interest due to contrast differences, regardless of their position, as there is no dependence in this respect.

With this purpose, the clinical specialist authors, using the interactive MATLAB [26] function that captures pixel coordinates on the images, manually select at least *n* ≥ 10 points on each edge, obtaining the corresponding coordinates (*x*_*i*_,*y*_*i*_), which are labeled as ground truth once they are agreed upon by all specialists. A polynomial of degree *d*, $\hat{y} = p_{1} x^{d} + p_{2} x^{d - 1} + ... + p_{d} x + p_{d + 1} ,$ is adjusted by applying least squares regression with the ground-truth points, with *d* varying from 1 to 3. A grade higher than three is not necessary, as the curvature of the screw edges is relatively smooth. The polynomial that best fits the selected points is determined by measuring the *mean squared error* (*MSE*) that determines differences between the predicted point values and the ground-truth points. The lower the MSE is, the better the model’s predictive accuracy.

In our experiments, with the 1394 images finally used for testing, we used *N* = 3206 screw edges by setting *d* = 1, 2 and 3 and computing the *MSE* for each edge. The best fit is selected with the minimum *MSE* value, which is cataloged as the ground truth for the corresponding screw edge. We verified that 7% of polynomials have been with *d* = 1 and 92% with *d* = 2. Only a portion of 1% corresponds to polynomials with *d* = 3. These results justify the choice of degree two during the segmentation process explained in the Materials and Methods section. Table 3 summarizes the results derived from the experiments to determine the degree of the polynomial as a function of the minimum value of *MSE*.

**Table 3:** Determination of the degree of the polynomial as a function of *R*^2^

*N* = 3206 (screw edges)			
*d*	1	2	3
*MSE*: Number of edges and percentage	224 (7%)	2949 (92%)	33 (1%)

Now, we compute a matching score for each screw edge (*ms*_*e*_) by comparing the ground-truth line (*G*) and the one adjusted (*A*) by the proposed procedure with *d* = 2 and *N* = 3206. In this regard, following the vertical direction of the image and, therefore, along the *y*-coordinates, we determine the minimum (*Y*_*m*_) and maximum (*Y*_*M*_)* y*-coordinates of the overlap between *G* and *A*. From *Y*_*m*_ to *Y*_*M*_ and for each *y*-coordinate (*Y*_i_), we obtain the corresponding *x*-coordinate value for *G* (*X*_*Gi*_) and *A* (*X*_*Ai*_) and compute the matching score as follows:

3 $$ms_{e} = \frac{{\sum\nolimits_{{i = Y_{m} }}^{{i = Y_{M} }} {\left( {X_{Gi} - X_{Ai} } \right)^{2} } }}{{Y_{M} - Y_{m} }}$$

Expression (3) represents the average deviation values between the *X*-coordinates for each edge (*e*). Considering the *N* tested edges, we compute the statistical average (*m*_*e*_) and standard deviation (*σ*_*e*_), both related to *m*_*se*_, obtaining 2.75 and 1.01 pixels, respectively.

To determine the validity and significance of the results for the proposed approach, we designed a testing strategy based on the one-sample statistical Student’s *t* distribution (*t* test), [39, 40] which is useful when a small number of samples is involved. The *t* test is recommended against the *z* test (which assumes a normal or Gaussian distribution) when the standard deviation of the population from which the samples are collected is unknown, as is the case in our experiments. The testing strategy is focused on each edge to be detected. The samples are defined through the statistical variable $x_{e} \equiv X_{Gi} - X_{Ai} ,$ i.e., each sample in *x*_*e*_ is the difference between the *X*-coordinates between the detected points (*A*) and the ground-truth points (*G*). The length of each edge (*L*_*e*_) is defined by the difference *L*_*e*_ = *Y*_*M*_ – *Y*_*m*_, which is the number of samples in *x*_*e*_. In our experiments, the *L*_*e*_ values are, on average, approximately 285, i.e., the dimensions of *x*_*e*_. The degree of freedom for this test [38, 39] is *df* = *L*_*e*_ –1, i.e., *df* > 30, which is a value accepted by the scientific statistical community.

The best performance is obtained when *x*_*e*_ values are null, a perfect match between the fitted edge line (*A*) and the corresponding ground-truth line (*G*). This defines Hypothesis *H*_1_ in the *t* test and alternative Hypothesis *H*_0_ (*x*_*e*_ values differ from zero). To measure the performance of these hypotheses, we use the *p* value [41] as the probability of obtaining results from the test at least as extreme as the observed results, assuming *H*_0_ is true. Low *p* values indicate that the extreme observed outcome is very unlikely under *H*_0_ (rejection of nonmatches) and specified by the significance level (*α*) set to 0.05 in our experiments, which is a typical value [42].

We tested the *N* edges individually by computing the *p* value for each edge, obtaining an averaged *p*_*p*_*-*value of 0.0106 over *N*, i.e., the degree of rejection of *H*_0_ is high. Only 2.43% of the *N* edges tested were not rejected. The estimated standard deviation averaged with the *N* edges (*σ*_*p*_), is 0.981 and, therefore, on the same order of magnitude as *σ*_*e*_ obtained above. According to these results, we can conclude that the deviations obtained against the ground truth are acceptable in terms of resolutions computed in millimeters. Table 4 summarizes the statistical results obtained for the *N* edges with a polynomial degree of *d* = 2.

**Table 4:** Statistical measures on fitting the lines along the screw edges from *ms*_*e*_

*N* = 3206 (screw edges), *d* = 2					
*Enhanced*	*m*_*e*_	*σ*_*e*_	*p*_*p*_*-*value (*α* = 0.05)	*σ*_*p*_	% *H*_*0*_ (*no rejected*)
*No*	2.75	1.010	0.0106	0.981	2.43
*Yes*	2.48	0.908	0.0101	0.887	2.19

**Fig. 10:**
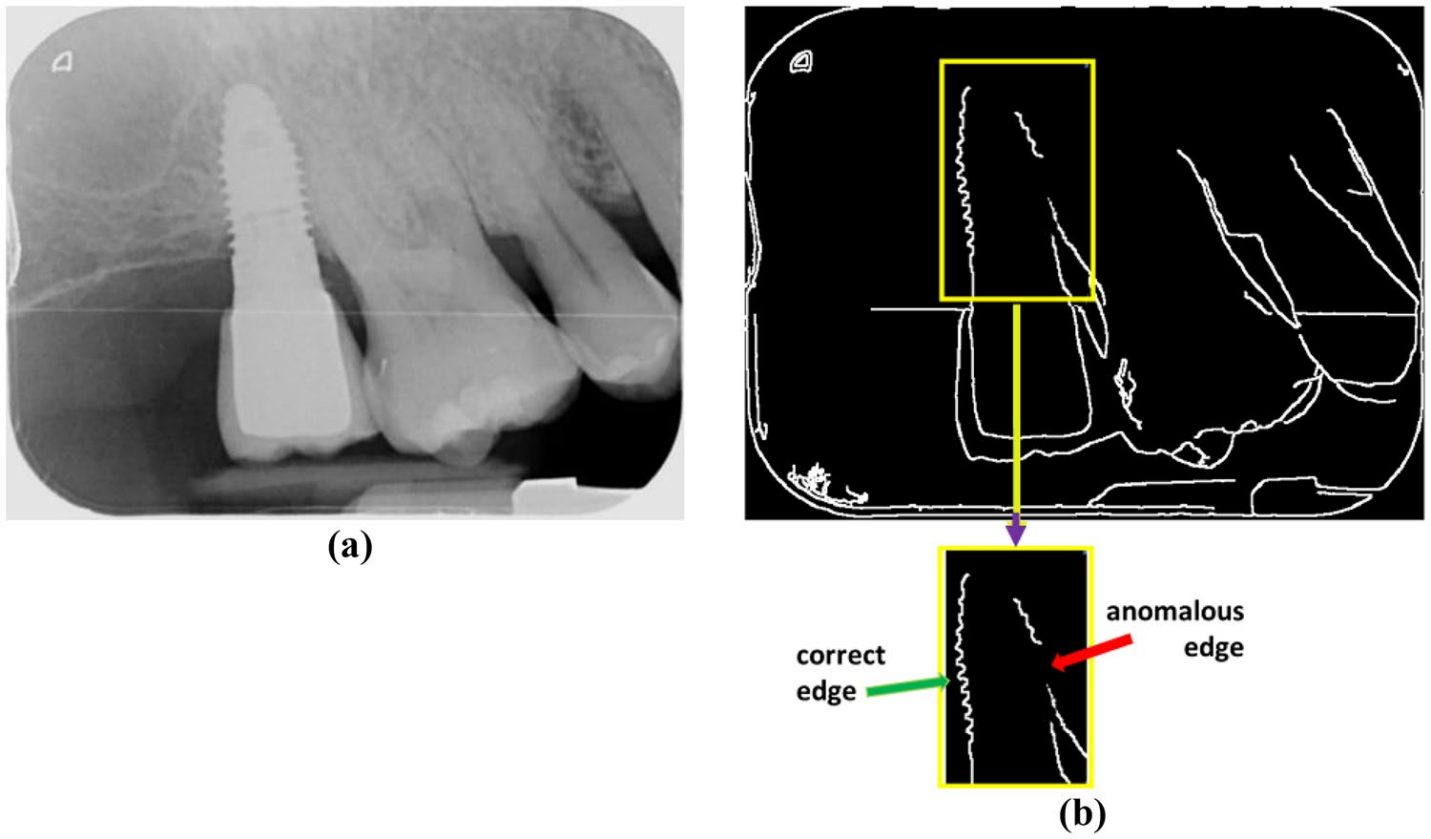
**a** Original image containing an implant with low contrast; **b** edge extraction with and without anomalies

The main errors in the process of line fitting along the screws come mainly from edge extraction due to the low contrast in intensity levels between the implant and the surrounding tissues. This causes the edges to appear broken with discontinuities and branching, resulting in incorrect fits. Figure 10 displays an illustrative example of this issue. Indeed, analyzing the image in Fig. 8a, reproduced in Fig. 10a on its original representation, an apparent lack of contrast between the implant and the surrounding tissues can be seen. Figure 10b displays the resulting edges after applying Step 1 in detecting the implant boundary section. The bottom part displays the borders inside the bounding box once it has been expanded by 10%, according to Step 2 in detecting the implant boundary section.

**Fig. 11:**
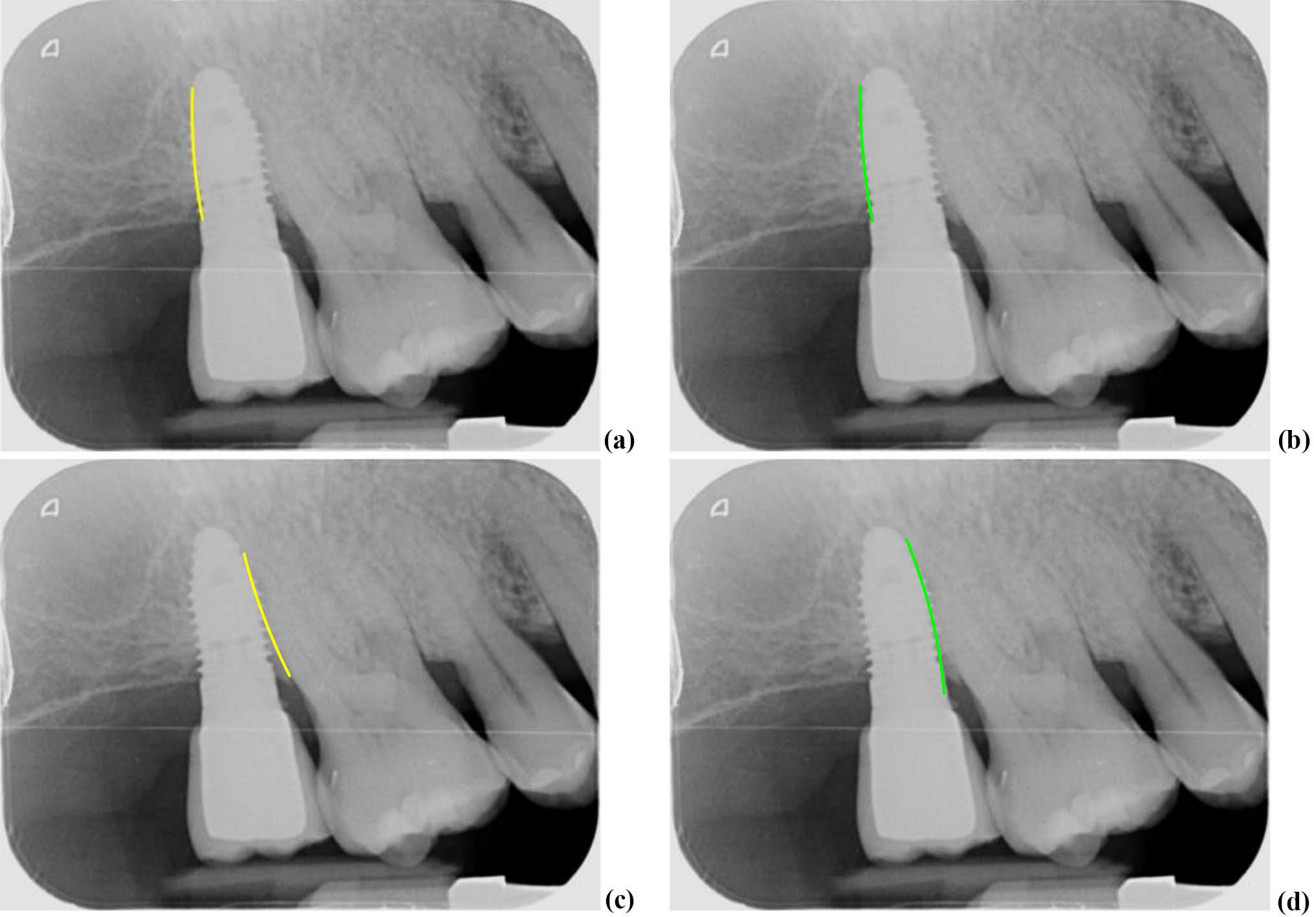
**a**–**b** Left edge correctly detected and its ground truth; **c**–**d** right edge incorrectly detected and its ground truth

The left edge of the screw appears correctly segmented, but the right side shows a clear anomaly. The result of this effect is that the left edge appears correctly detected, Fig. 11a yellow line, or at least with minimal deviations from its corresponding ground truth, Fig. 11b green line, while the detection of the right edge, Fig. 11c yellow line, appears erroneously detected with respect to its ground truth, Fig. 11d green line. In conclusion, it is worth mentioning that the null hypothesis was rejected in the first case and not rejected in the second, which allows us to clearly identify the source and origin of the errors made in the adjustment of the lines to the screws.

**Fig. 12:**
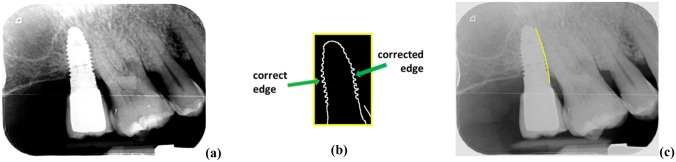
**a** Enhanced image; **b** corrected edge with Canny; **c** right edge correctly detected

To address this problem, the 1460 images dedicated to testing the *IU* process have been all enhanced by applying a linear transformation to each input image so that the intensity values in the range [*m*-*σ*, *m* + *σ*] are mapped to the range [0, 255], where *m* and *σ* are the mean intensity value of the input image and the standard deviation, respectively. Continuing with the illustrative example above, Fig. 12a displays the enhanced image; in (*b*) the edges extracted with Canny, where both edges were correctly detected; in (*c*) the right edge correctly detected. With this new test, an improvement of approximately 9% was achieved in all the results shown in Table 4 compared to the results obtained with the same images without enhancement. The degree of rejection of *H*_0_ has improved, with 2.19% of the *N* edges tested not rejected. The Canny edge extractor (containing smoothing, gradient, nonmaximum suppression and hysteresis thresholding) is sufficiently robust against noise and other artifacts in the images, outperforming other edge extractors, hence its usefulness in this type of image. Furthermore, it is not affected by the relative arrangement of the implants in the upper or lower jaw.

#### Bone Resorption in Terms of Percentage

The last analysis is focused on the measurement of the error obtained when computing the percentages of bone resorption based on the identification of the significant points. Again, the clinical authors, using the interactive function from MATLAB [26] that captures pixel coordinates on the images, manually mark and agree on the two significant points (ground-truth points) on each ground-truth edge. Traversing the edge between the two manually marked critical points, we compute the piece of each ground-truth line and its length. The ratio between this length and the full length of the corresponding ground-truth line determines what is considered the correct percentage of bone resorption. This process is identical to the one proposed for the *IU* method, described in detecting implant boundary sections, but now in relation to the ground truth. Therefore, for each fitted line to each edge of the screws, we obtain two percentage values, one (*A*) provided by the proposed *IU* method and another (*G*) from the ground truth. The difference between the two percentages is defined as the error: *E* = *A* − *G*.

The 1394 images used for testing, without enhancement, are grouped into ten batches, with approximately 140 images per batch and a number of screw edges determined by the number of implants with their edges, making up a number of *E* percentage differences varying approximately from 340 to 400. If a significant point is not detected with the *IU* approach, the corresponding error associated with the ground truth is not computed. Therefore, for each batch, we build the variable *x*_*b*_ of observations with *E* as samples. On this variable *x*_*b*_, we also apply a *t* test and define Hypothesis *H*_1_ as follows: *“the mean of the differences between the percentages are zero,* i.e.*, both percentages A and G are identical, null error E”*. The alternative hypothesis is *H*_0_, expressing that *A* and *G* are different with a nonzero error *E*. We compute the *p* value for each batch and obtain an averaged *p*_*b*_*-*value of 0.0213 over the ten batches, also with the significance level (*α*) set to 0.05. The estimated statistical mean (*m*_*b*_) and standard deviation (*σ*_*b*_) of the errors (*E*) averaged over the ten batches were 2.63 and 1.28 pixels, respectively. Table 5 summarizes the statistical results of the bone resorption process. Because the *p*_*b*_*-*value is less than 0.05, the test is statistically significant. Therefore, from a clinical point of view, we can infer that our automatic approach is able to accurately detect bone loss due to peri-implantitis when it exists and as a direct consequence of an implant. This allows specialists to be aware of the problem, which is particularly relevant in the early stages for possible prevention or in later stages for tracking and therapy. Additionally, considering that the average lengths of implants are approximately 10 mm and that with a radiograph of the type used in our experiments, this same length is equivalent to approximately 160 pixels in the vertical axis, the average error represented by *m*_*b*_ is approximately 0.17 mm with *σ*_*b*_ of approximately 0.08 mm. Thus, according to the study in [43], if the average annual bone loss is approximately 0.4 mm, with these errors, we are in a very good position for early bone loss detection. With respect to the strategies [8] and [20] mentioned in the introduction and Table 1, with the same purpose, it should be noted that they both use deep learning–based object detectors, Mask R-CNN and Faster R-CNNN, to extract the key points from the feature maps in convolutional layers, while we also use an object detector (YOLOv3) in the *DL* part only to roughly locate the crowns and screws and then the process described in *IU* for the precise identification of the significant points. This is why the identification of the required points of interest from the feature maps provided by the convolutional layers was not successful in our radiographs.

**Table 5:** Statistical measures for bone resorption

*No. of images* = 1394; *No. of batches* = 10		
*p*_*b*_*-*value (*α* = 0.05)	*m*_*b*_	*σ*_*b*_
0.0213	2.63	1.28

During the detection of the significant points, we identified two clear sources of error. When there is no or very little apparent level of bone resorption and when the transition in the intensity profile is not abrupt but gradual, both cases were related to the process described in Step 3 of detecting the implant boundary section. The first source of error appeared when bone resorption was nonexistent (or normal with low impact), and the procedure simply failed to detect the significant points; see Fig. 8a as an illustrative example of this situation. This is because according to the procedure described in detecting the implant boundary section, Step 3, no abrupt changes in intensity levels appeared due to tissue degradation, and therefore, no change in slope in the intensity profile is identified, as illustrated in Fig. 6. In these cases, the significant points are not identified without affecting rejection or acceptance of the null Hypothesis *H*_0_. From a clinical point of view, this is not very problematic due to the absence of bone loss.

**Fig. 13:**
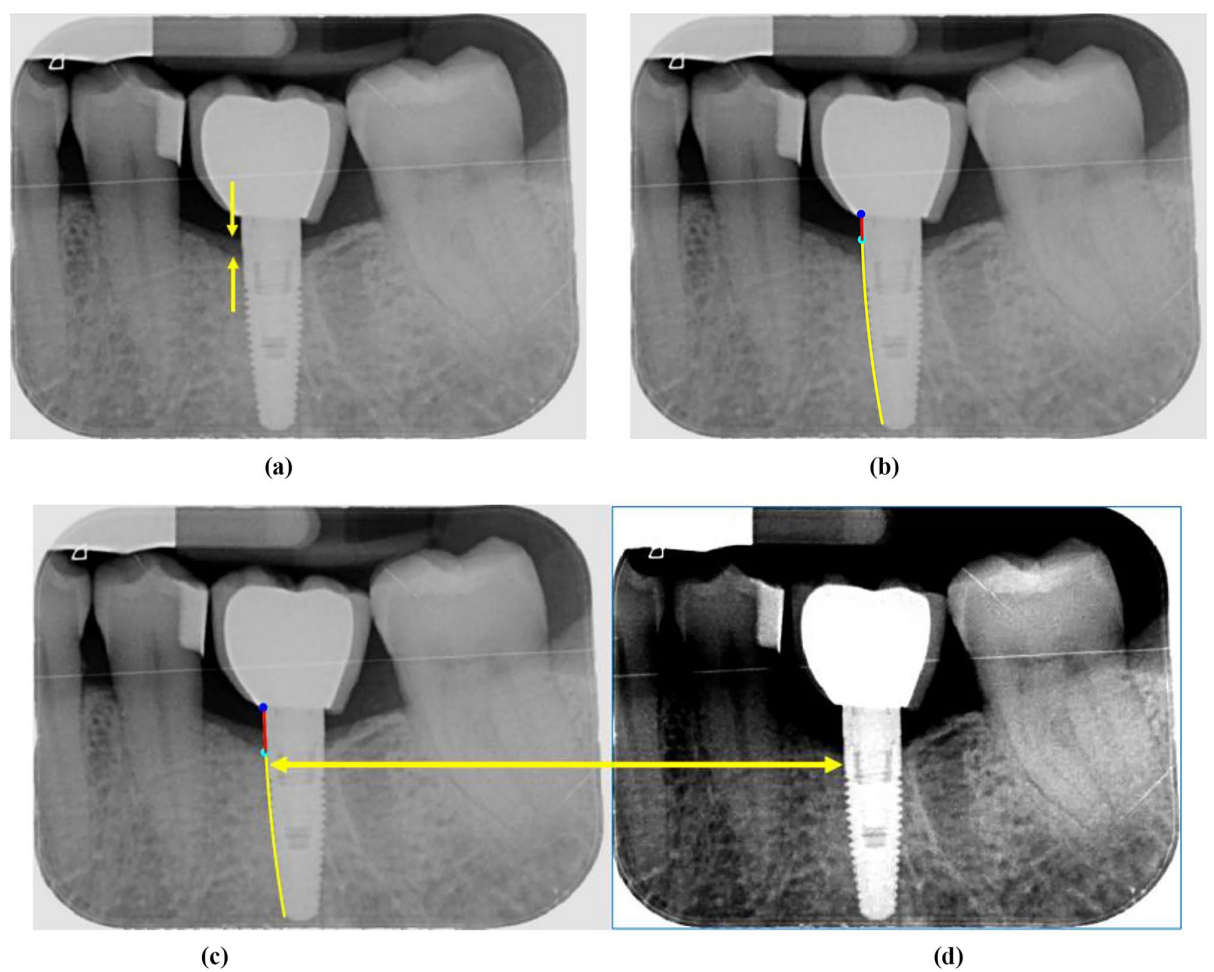
**a** Original image with a smooth transition along both edges (heads of arrows); **b** incorrect critical point detection; **c** ground-truth critical point; **d** enhanced image and critical point position (right head of the arrow)

The second case arises due to smooth transitions of the intensity level along both edges in the damaged area up to the dark area. This is because the tooth structure, including the tissue, is three-dimensional (3D) and the image is two-dimensional (2D), i.e., this results in a loss of existing information in the 3D structure when mapped onto the 2D image. The heads of arrows in Fig. 13a illustrate this situation. The identification of the significant point corresponding to the transition is incorrectly identified, Fig. 13b, against what is a correct detection (ground truth), Fig. 13c. If the same enhancement is applied to the image, as previously indicated in Fig. 13d, the doubtful transition zone disappears, but a larger displacement than the real one is produced, as shown by the double arrow between Figures (c) and (d), also causing an erroneous detection of the critical point. During testing, these misdetections clearly contribute to the nonrejection of the null Hypothesis *H*_0_. From a clinical point of view, given that there is bone loss, there is detection even if the extent of degradation is not exactly what is desired. The method for correcting this error is to place the patient’s jaw in the correct position in front of the capture device so that the 3D part is aligned in the image.

## Conclusions

We designed an automatic strategy by applying *AI* methods to determine the degree of bone resorption involving soft and hard tissues, with bone loss, in intraoral radiographs due to inflammation produced by the implants in what is known as peri-implantitis. The method is a direct translation of human reasoning, i.e., under the paradigm of computational methods in *AI*. A promising area in continuous growth and ongoing development in odontology [11]. Intraoral radiographs, with their associated complexity displaying different intensity levels and structures, are the basis of this study. The automatic process was designed with different stages or levels of progress (*DL* and *IU*). At the *DL* level, the YOLOv3 deep learning–based object detector demonstrated its ability to detect crowns and screws on implants assuming an important contribution in the initial phase of the strategy. This allows the posterior techniques, integrated into the *IU* level and based on computer vision techniques, to be applied conveniently to identify the edges of the screws and significant points along such edges and thus the areas damaged by peri-implantitis, with quantification, in terms of percentage, with respect to the full length of the screw. This allows the dentist to identify the degree of bone resorption since the dentist knows in advance the dimensions of implants, which represents highly relevant assistance for the diagnosis of the severity of peri-implantitis. The results obtained are completely acceptable for the dataset of images analyzed from computational and clinical points of view.

As an additional conclusion, it is worth mentioning that the automatic procedure can be generalized to other populations whose periapical radiographs have similar characteristics, even if they are obtained with a different device. In this case, an adaptation in the *DL* process to relabel the bounding boxes (crowns and screws) involved during training would be desirable. However, it is still possible to keep the approach used in our experiments and resize the images to the required dimensions in pixels (410 × 340). The *IU* process is perfectly applicable as it is defined, except for the fact that device calibration would be needed to establish the equivalence between pixels and millimeters.
